# Editorial: Eicosanoids and cytokines: Resolution of inflammation

**DOI:** 10.3389/fphar.2022.978331

**Published:** 2022-08-11

**Authors:** Jun-Yan Liu

**Affiliations:** Center for Novel Target and Therapeutic Intervention, Institute of Life Sciences, Chongqing Medical University, Chongqing, China

**Keywords:** eicosanoid, cytokine, inflammation, polyunsaturated fatty acid, cyclooxygenase (COX), Cytochrome P450 (CYP), lipoxygenase

Eicosanoids and cytokines constitute an interactive network, playing pivotal roles in the onset, development, and resolution of inflammation. Many eicosanoids are pro-inflammatory mediators, including but not limited to prostaglandin E_2_ (PGE_2_), leukotriene B_4_ (LTB_4_), and 20- hydroxyeicosatetraenoic acid (20-HETE), representing the metabolites of primary metabolic pathways of cyclooxygenases (COXs), 5-lipoxygenase (5-LOX), and cytochrome P450 (CYP) ω-hydrolases, respectively. In contrast, some eicosanoids are anti-inflammatory mediators, such as resolvins (Rv), protectins (PD), maresins (Ma), and lipoxins (LX), as well as epoxyeicosatrienoic acids (EETs), which are derived from arachidonic acid (AA), eicosapentaenoic acid (EPA), and docosahexaenoic acid (DHA) in the presence of LOXs, COXs, and CYP monooxygenases. However, it should also be noted that the role of resolvins and lipoxins as anti-inflammatory endogenous mediator were challenged by Schebb et al. (2022) by comprehensively reviewing the formation, signaling and occurrence of resolvins and lipoxins. An ultra-sensitive quantitative method that could quantity the levels of most resolvins and lipoxins in biological samples is urged to provide more evidence to resolution of this controversy. Recently, more attention has been paid to investigating the functions and underlying mechanisms of the metabolites of ω-3 polyunsaturated fatty acids (PUFAs) such as DHA and EPA, instead of DHA and EPA themselves and the metabolites of ω-6 PUFA such as AA and linoleic acid (LA). These metabolites include maresins, protectins, maresins, resolvins, hydroxydocosahexaenoic acids (HDoHE), hydroxyeicosapentaenoic acids (HEPE), epoxyeicosatetreaenoic acids (EEQ), epoxydocosapentaenoic acids (EDP), and many others (Leuti et al.; Ni and Liu). In addition, more attention was paid to the complicated metabolite network. An eicosanoid mediator is a metabolite mediated by one enzyme and also a substrate of another enzyme. For example, both 5-LOX-mediated metabolite LTB_4_ and COX-mediated PGE_2_ are the substrates of prostaglandin reductase 1 (PTGR1), a therapeutic target for cancers Wang et al. Again, 20-HETE, as its substrate AA, is also a substrate of COXs that could form 20-OH-PGE_2_ Ni and Liu.

Cytokines, including but not limited to chemokines, interferons, interleukins, lymphokines, and tumor necrosis factors, mediate the inflammatory process by modulating multi signaling pathways. Erythropoietin (EPO), a glycoprotein cytokine secreted by the kidney and fetal liver, has therapeutic effects in neurodegenerative diseases and ischemic stroke by binding to EPO receptors (EPORs) Ma et al. IL-1β and IL-18 have been reported to get involved in the attenuation of seizures severity modulated by inhibition of gasdermin D-mediated pyroptosis in a kainic acid-induced epileptic murine model Xia et al.

Rising interests in the interaction between cytokines and eicosanoids resulted in controversial findings on the causative effects between cytokines and eicosanoids. In contrast, a feedback interplay between eicosanoids and cytokines was found in many inflamed and resolution statuses. We believe manipulation of eicosanoid and cytokine storms is still a key therapeutic strategy for the resolution of inflammation.
